# The Evolution of Reverse Total Shoulder Arthroplasty—Where Do We Stand and What Comes Next?

**DOI:** 10.3390/jcm12051945

**Published:** 2023-03-01

**Authors:** Stefan Bauer, Lukas Ernstbrunner

**Affiliations:** 1 Service d’Orthopédie et de Traumatologie, Chirurgie de l’Épaule, Ensemble Hospitalier de la Côte, 1110 Morges, Switzerland; 2 Medical School, University of Western Australia, 35 Sterling Highway, Perth, WA 6009, Australia; 3 Department of Orthopaedic Surgery, Royal Melbourne Hospital, 300 Grattan Street, Parkville, Melbourne, VIC 3050, Australia; 4 Department of Biomedical Engineering, University of Melbourne, Parkville, VIC 3010, Australia

**Keywords:** reverse total shoulder arthroplasty, history, biomechanics, lateralization, implant design, mixed reality

Over 35 years ago, the pioneer Paul Grammont from Lyon published his ideas of a reversed semi-constraint prosthesis improving the moment arm of the deltoid by medializing the center of rotation and lengthening of the arm and thus increasing deltoid muscle tension [1]. The original Grammont reverse total shoulder arthroplasty (RTSA) has undergone remarkable design improvements, thus not only relieving pain, but also reliably restoring shoulder “balance” and function especially for activities that require an upper limb that needs to be held and stabilized in space against gravity and weight [2,3]. These improvements include variable design configurations and glenoid-sided, humeral-sided and global lateralization with advantages such as reduced notching, increased range of motion and rotation, increased stability due to deltoid “wrapping” and higher joint reaction forces in elevated arm positions [4,5,6,7,8].

Due to the success of RTSA, indications have been expanded from irreparable cuff tears with and without osteoarthritis [9,10,11,12,13] to eccentric glenohumeral OA [14], rheumatoid arthritis [15], and proximal humeral fractures and their sequelae [16,17,18]. There are also advocates for RTSA over anatomic total shoulder arthroplasty (TSA) in concentric OA in elderly patients, and this appears justifiable since clinical results, patient satisfaction, and complications after RTSA and TSA in such patients do not show a significant difference with the exception of internal and external rotation in favor of TSA [19,20]. The increased utilization of RTSA is also displayed in the statistics of the largest worldwide shoulder arthroplasty registry, the Australian Joint Replacement Registry, including over 60,000 shoulder replacements implanted in 2021, of which around 80% were RTSA [21].

Remarkable developments around RTSA are available on the market including new preoperative planning software [22], augmented and mixed reality integration [23,24,25], and improved implant designs [4,5,26]. Together with increased surgical experience and knowledge about long-term results [11,27], it is thought that our results have become more reliable. However, at the same time, an increased number of RTSA-specific complications and failures are inevitable and complications are more frequently seen [28]. They include scapular notching [6,29,30], acromial and scapular spine stress fractures [31,32,33,34,35,36], humeral-sided and glenoid bone loss [37,38], instability [16,39], deltoid paralysis [40], and failure to restore sufficient internal [41,42] and external rotation [16,42]. Some of these complications can make revision surgery extremely challenging, requiring revision-specific preoperative planning tools [25], complex glenoid bone grafting procedures [43], or even custom-made implants [38].

Besides this burden, we are at the brink of integrating artificial intelligence data into our planning process and using mixed reality tools and robotics for more precise execution of our surgical plans, and we have been starting to integrate the soft tissues [44] as well as scapulothoracic biomechanics [8] into our surgical decision making. Artificial intelligence, patient-specific implantation and implants, mixed reality, robotics, muscle-tendon and tissue planning, and integration of scapulothoracic motion will be some of the key elements of the journey of RTSA in the future.

The aim of this Special Issue is a comprehensive review of the literature on indications, the evolution of RTSA implant designs, biomechanical considerations, current trends and design specific outcomes, discussion and provision of evidence of potential benefits of preoperative planning tools, and to shed light on unsolved problems for ongoing study and optimization of RTSA in the future. 
